# Supplementary figures and images for: Fusarium graminearum DICER-like-dependent sRNAs are required for the suppression of host immune genes and full virulence
Source: PLoS One. 2021 Aug 5;16(8):e0252365. doi: 10.1371/journal.pone.0252365 (PMC8341482; doi:10.1371/journal.pone.0252365)

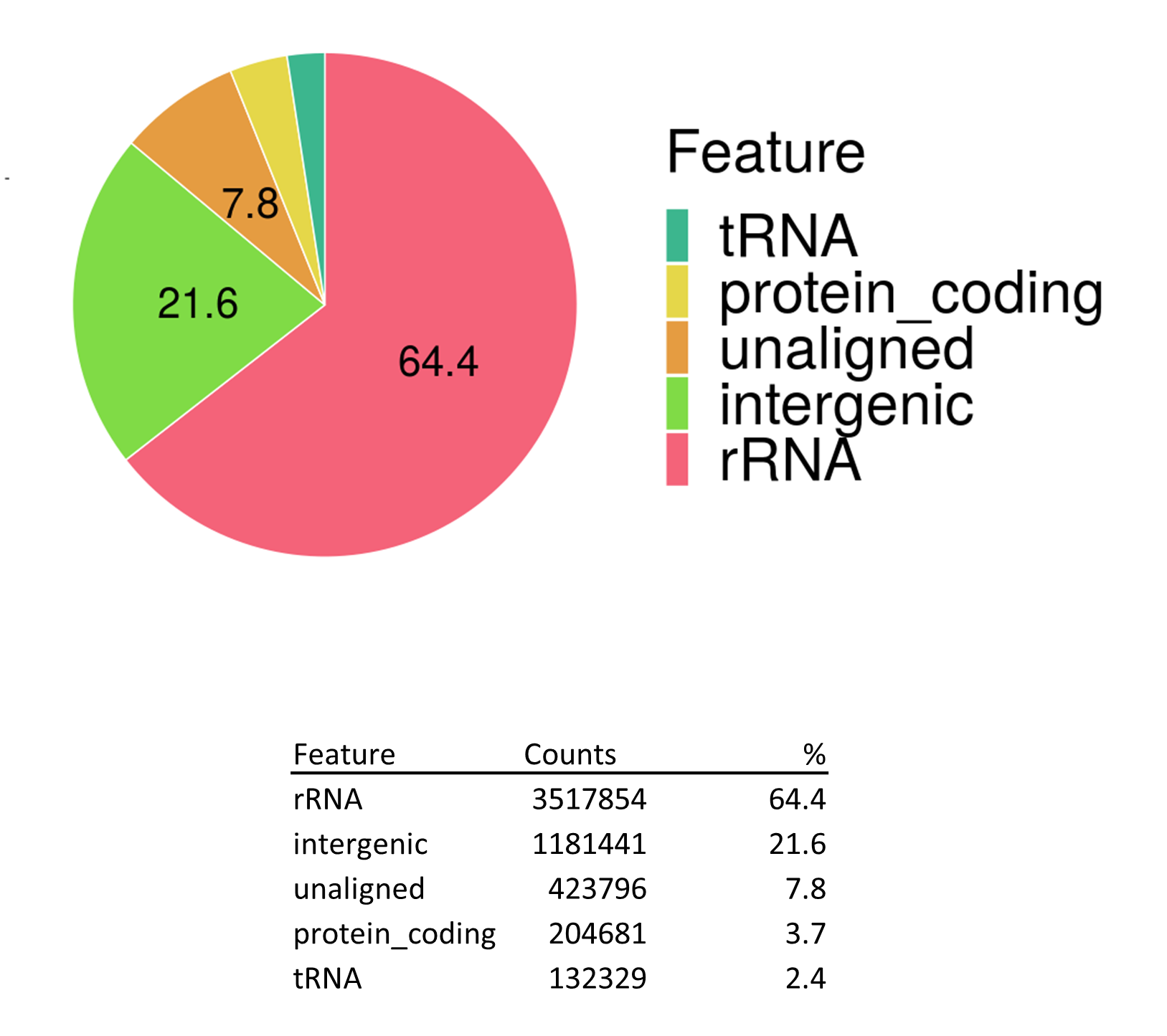

Supplement: S1 Fig — Reads were trimmed as described earlier and aligned to the PH1 reference genome (GCF_000240135.3_ASM24013v3) with bowtie2 [75]. (TIF) [file pone.0252365.s001.tif]

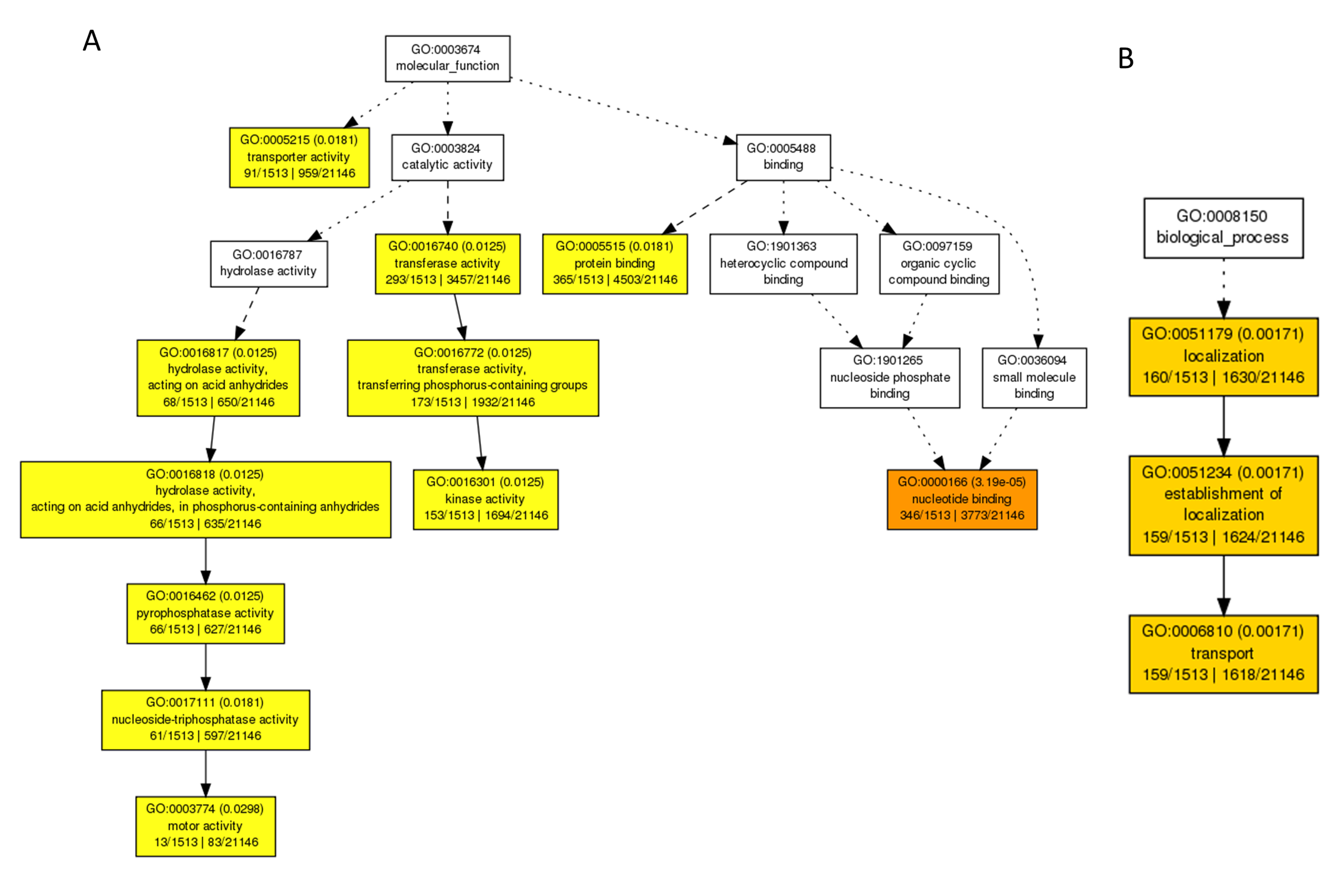

Supplement: S2 Fig — The plot shows all significantly enriched GO-terms in the target gene set for (A) molecular function and (B) biological process. The analysis was done using agriGo v2.0. Each box contains information regarding one term. GO: indicates the GO accession, in brackets the p-value is stated (Fisher; Yekutieli (FDR)). After the bracket the GO-term description is written followed by the number of genes associated with said term 1. in the gene set and 2. In the background. (TIF) [file pone.0252365.s002.tif]

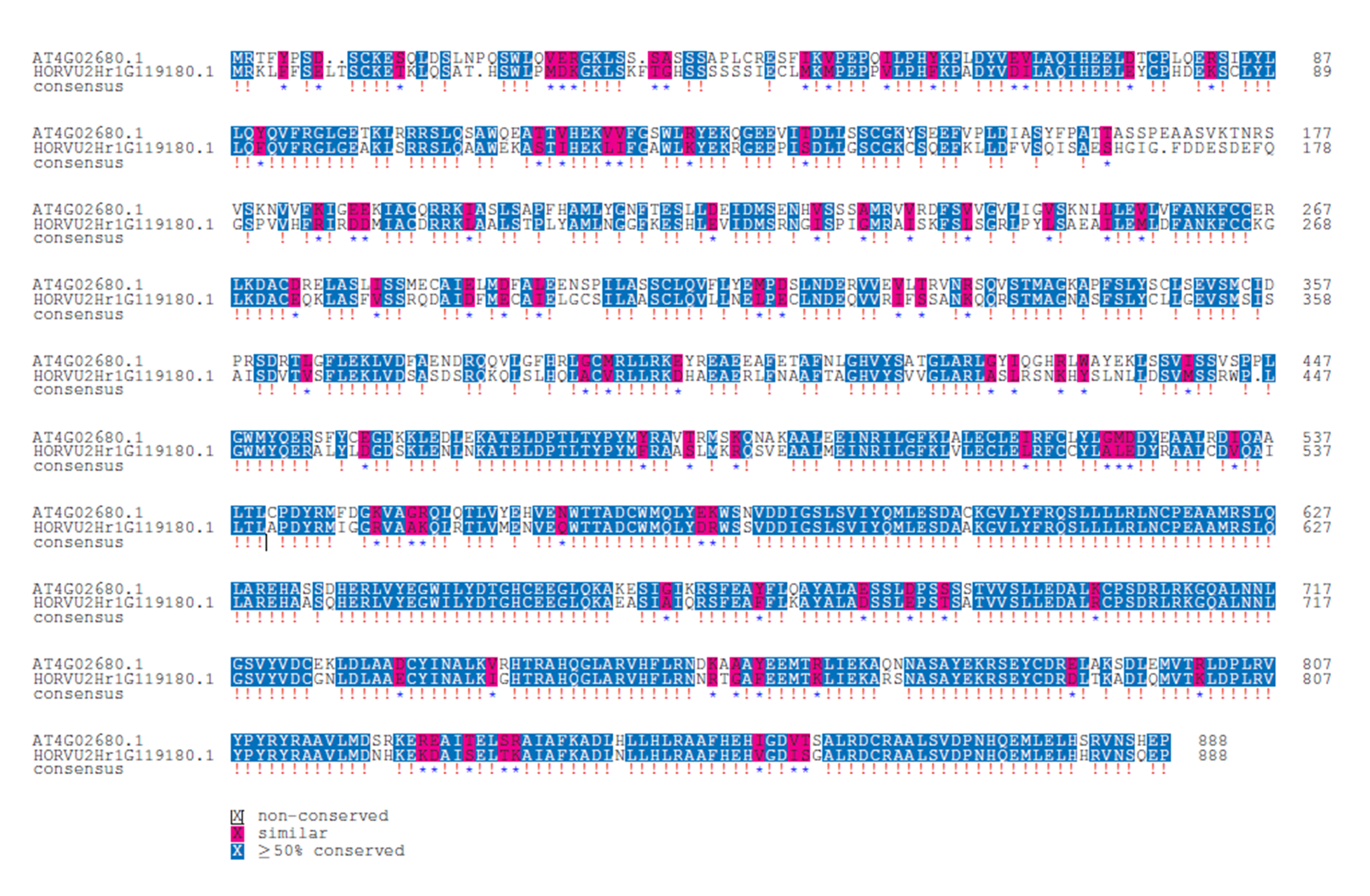

Supplement: S3 Fig — Identical amino acids are marked blue and similar amino acids are marked red. The alignment and visualization was done with the msa package for R [88]. (TIF) [file pone.0252365.s003.tif]

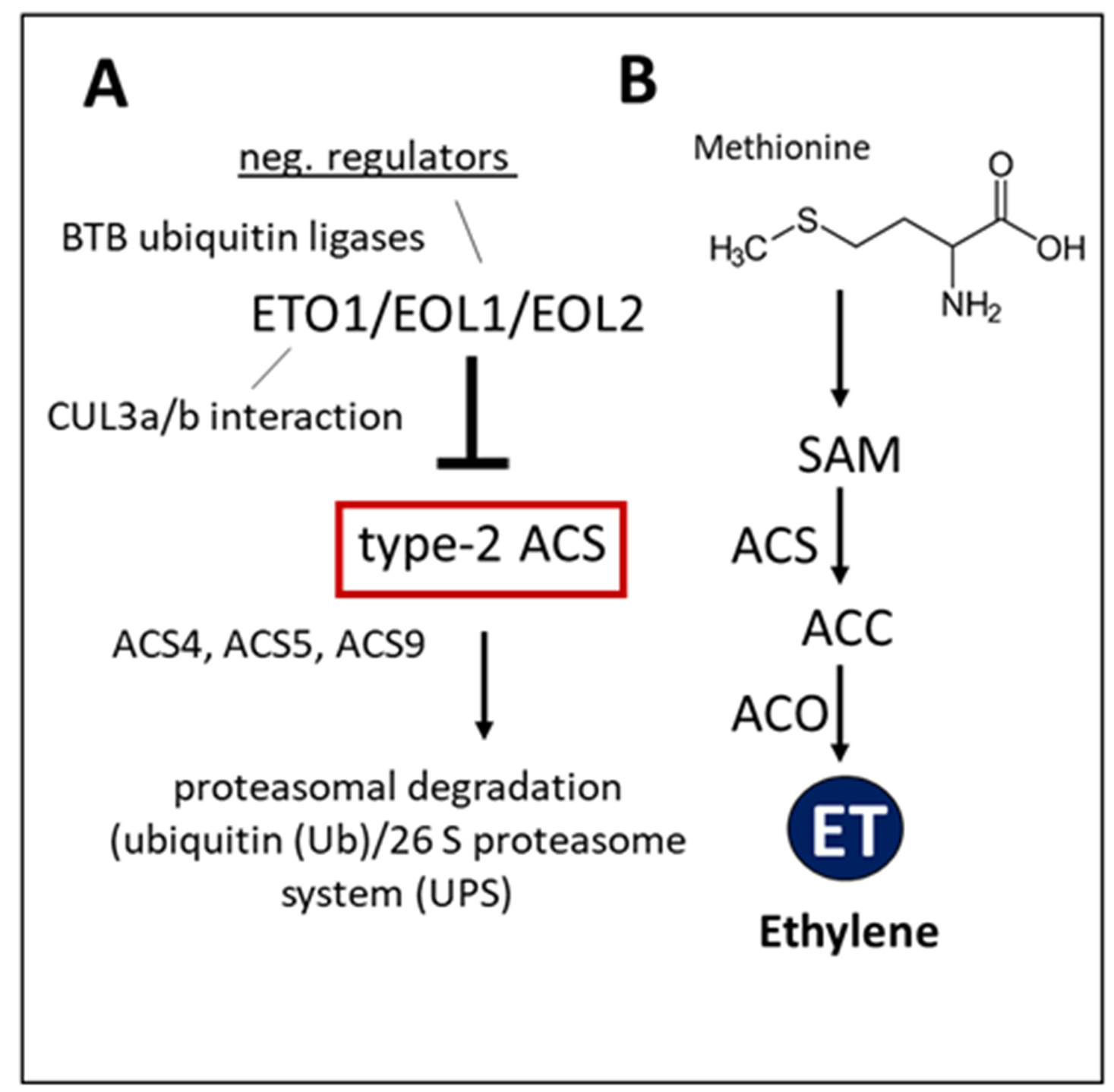

Supplement: S4 Fig — AtETO1 negatively regulates ethylene (ET) synthesis in At. AtETO1 acts together with AtEOL1 and AtETO1-like 2 (EOL2) in directing the ubiquitination and subsequent degradation of type-2 1-aminocyclopropane-1-carboxylate synthase (ACS) proteins (e.g. ET overproducer 2 (ETO2)), which produce the direct precursor of ET. (TIF) [file pone.0252365.s004.tif]

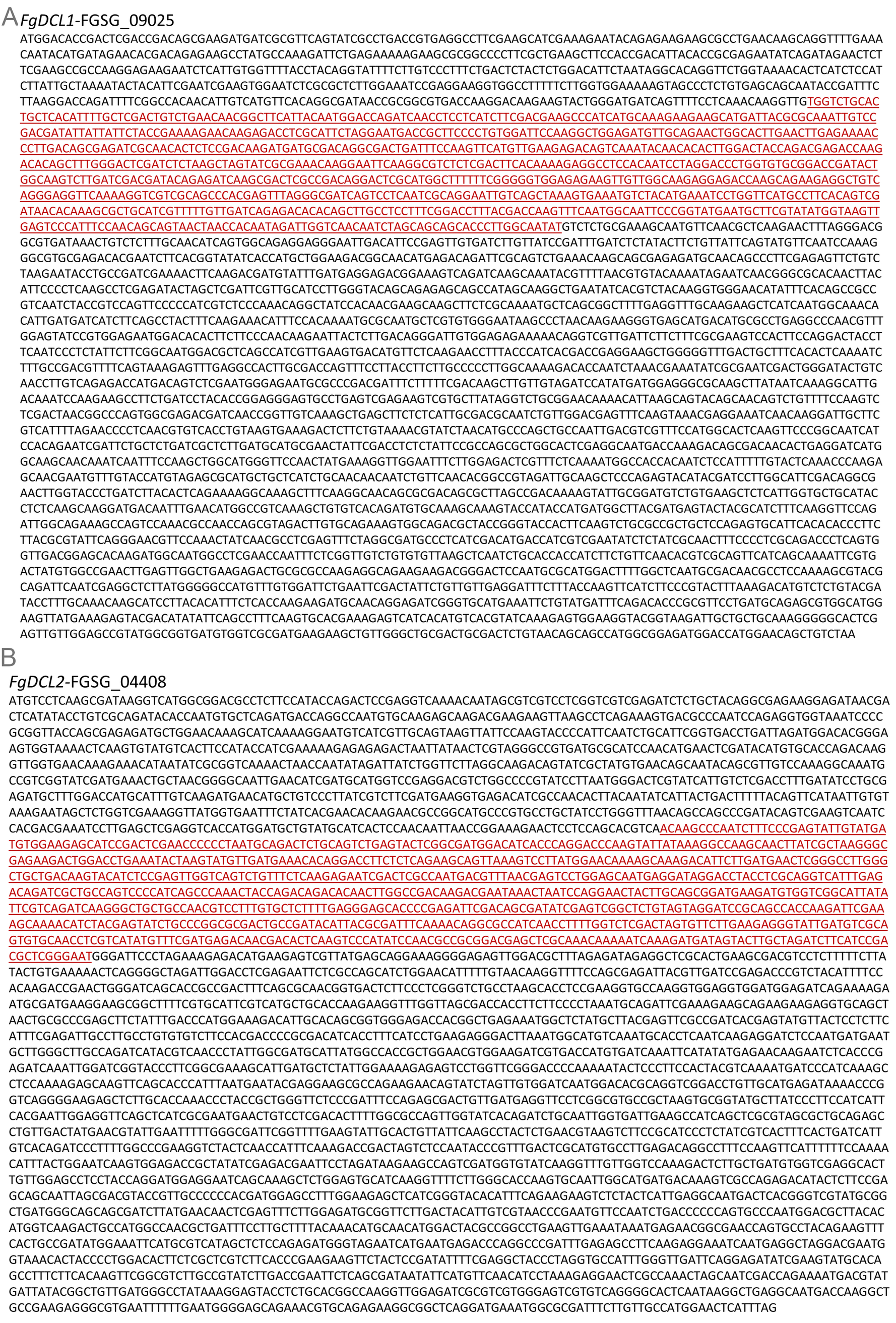

Supplement: S5 Fig — Coding Sequences (CDS) of the respective FgDCL genes with the sequences comprising the dsRNAs marked in red. A. FgDCL1-FGSG_09025 (912 nt long dsRNA-FgDCL1). B. FgDCL2-FGSG_04408 (870 nt long dsRNA-FgDCL2). (TIF) [file pone.0252365.s005.tif]

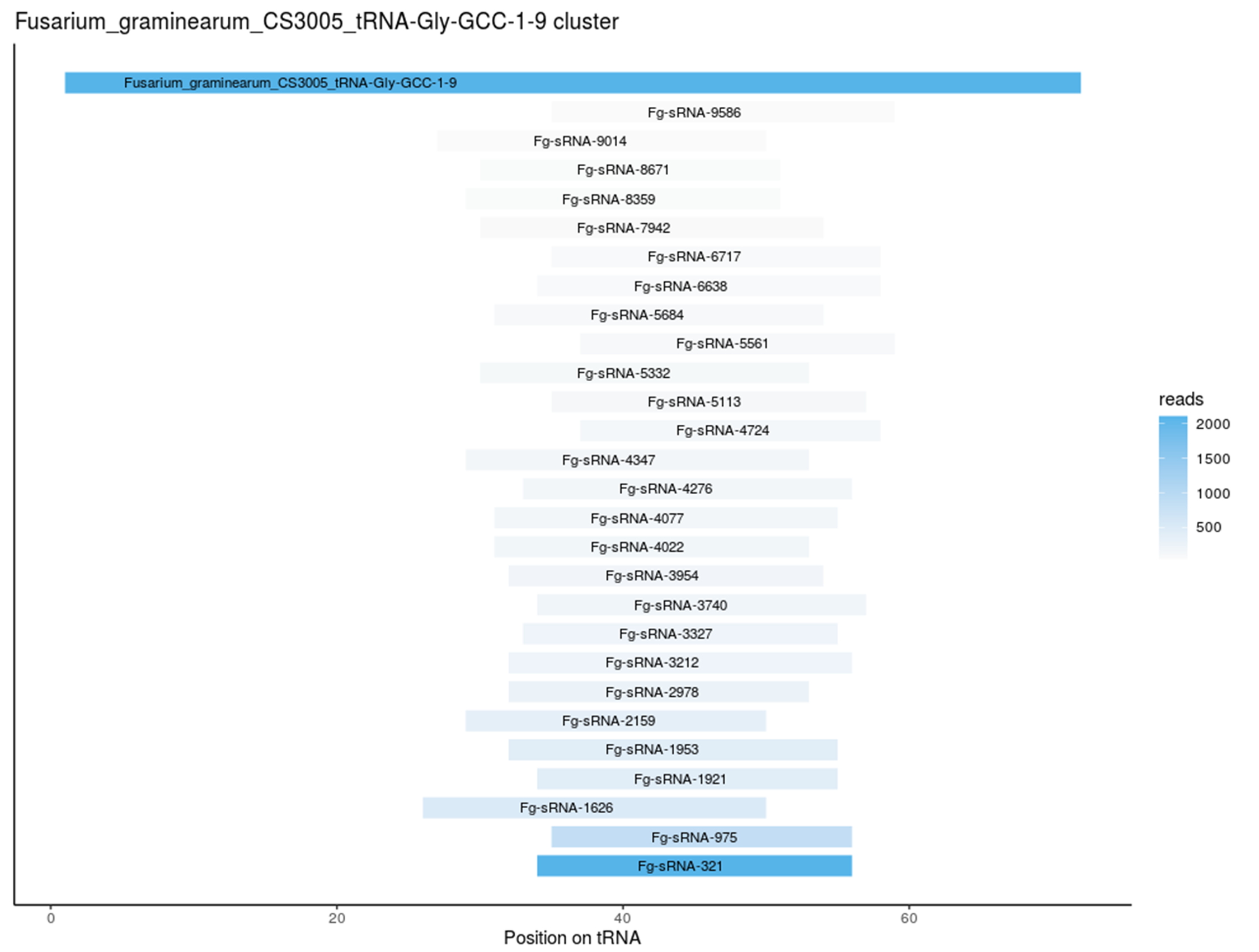

Supplement: S6 Fig — Alignment position of all Fg-sRNAs from axenic culture with more than 50 reads perfectly matching the Fg-tRNA-Gly(GCC)-9 gene (Fusarium_graminearum_CS3005-tRNA-Gly-GCC-1-9) colored by read count. (TIF) [file pone.0252365.s006.tif]

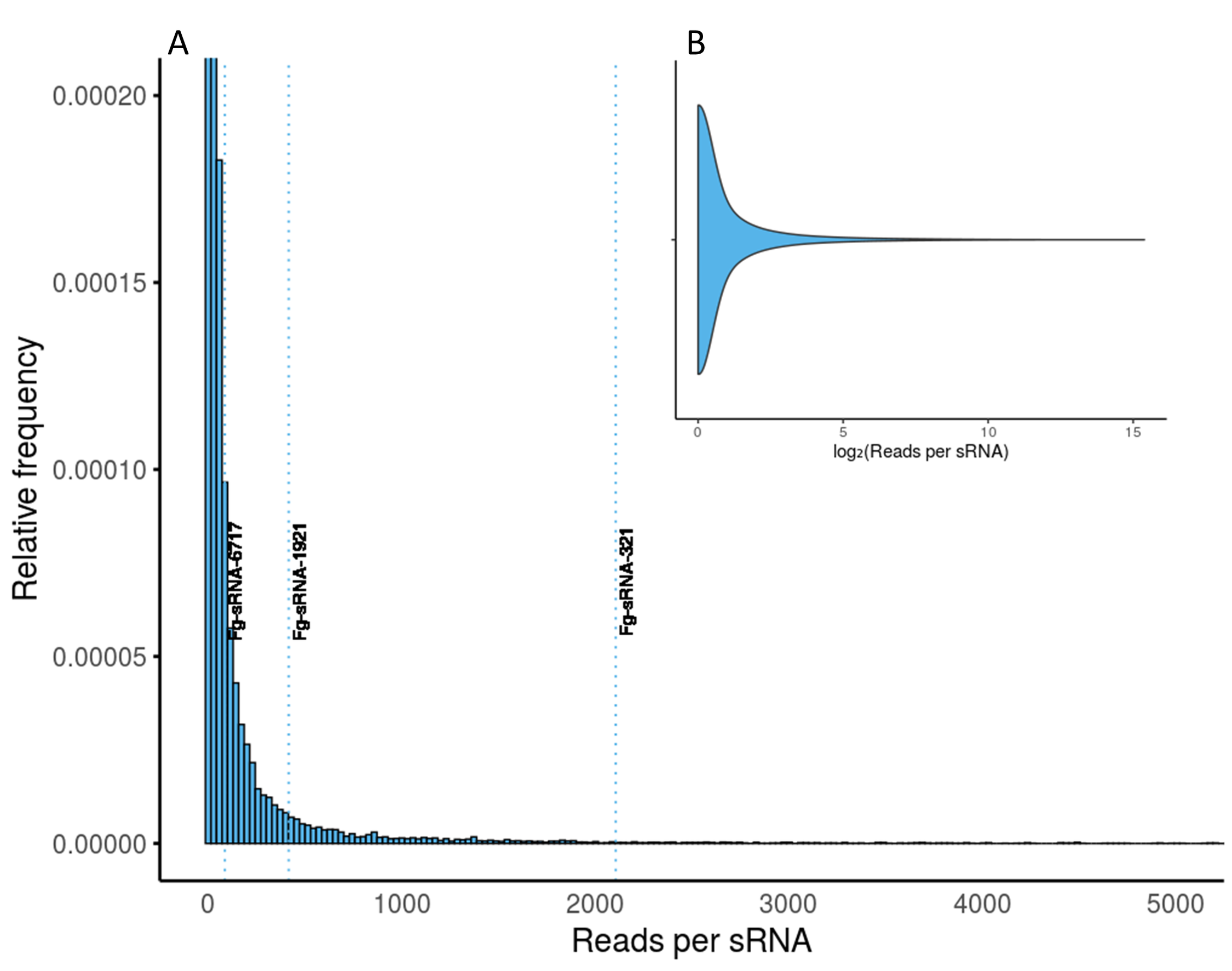

Supplement: S7 Fig — A: Histogram of the read count of every unique sRNA. The plot is truncated to make abundances recognizable. Most sRNAs have very low read counts and very few sRNAs have more reads than 3,000. Maximum read count per sRNA is 42,866. B: Violin plot of log2-transformed reads counts untruncated. (TIF) [file pone.0252365.s007.tif]

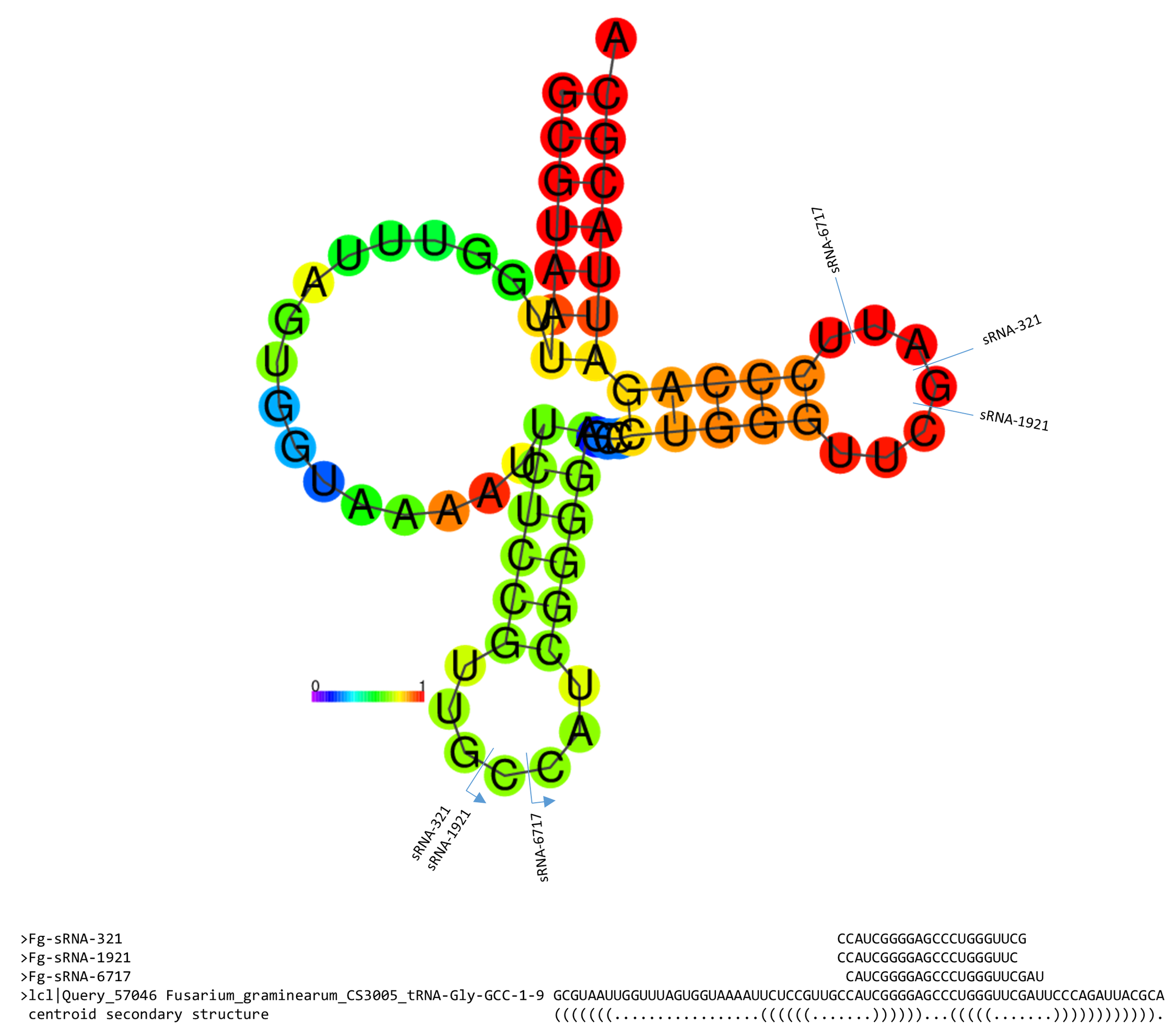

Supplement: S8 Fig — The centroid secondary structure of the Fg-tRNA-Gly(GCC) generated on the RNAfold web server (http://rna.tbi.univie.ac.at/cgi-bin/RNAWebSuite/RNAfold.cgi) with the origin and alignment of Fg-sRNA-321, Fg-sRNA-1921 and Fg-sRNA-6717. The colors of bases indicate the base pair probabilities. (TIF) [file pone.0252365.s008.tif]

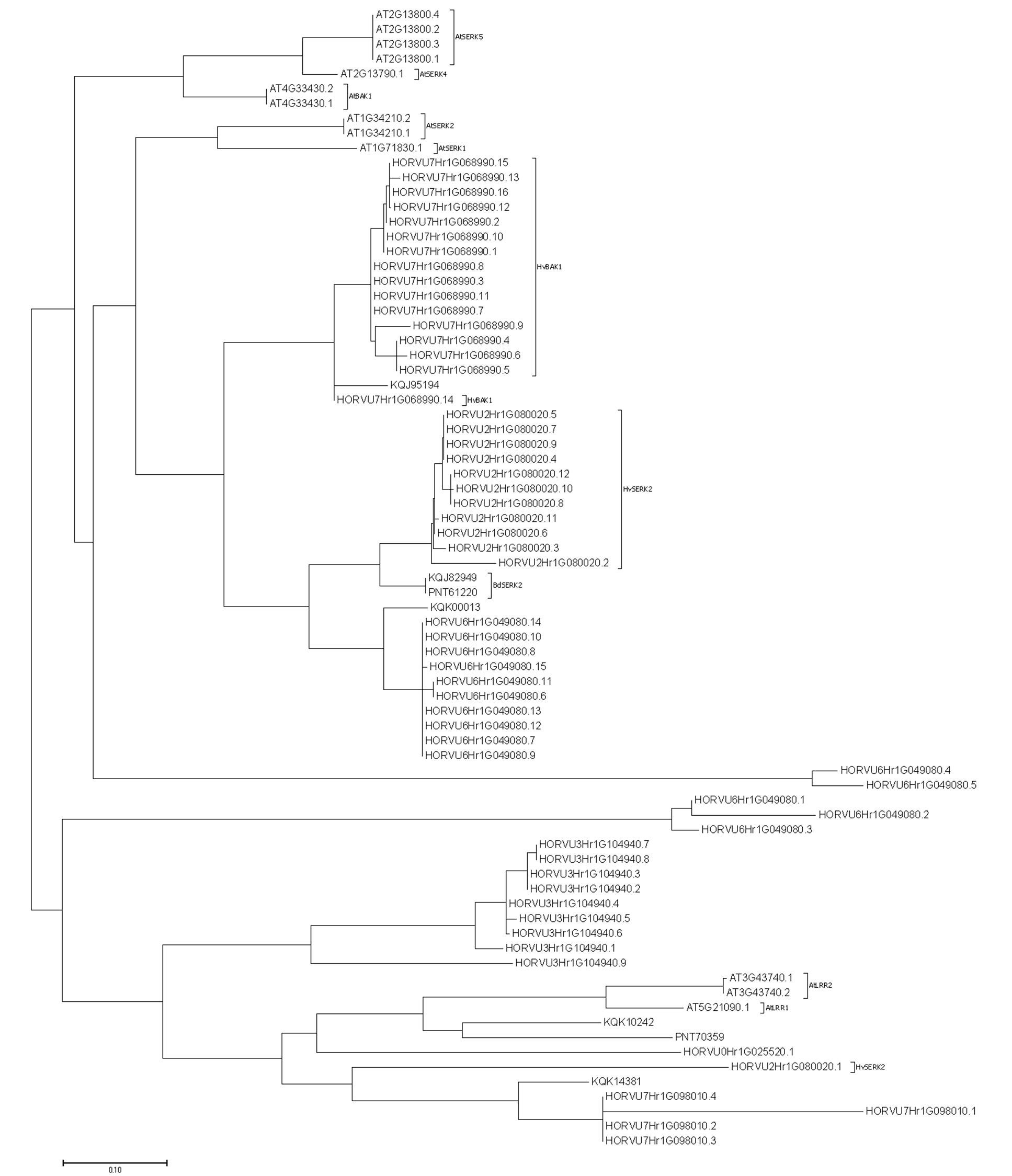

Supplement: S9 Fig — The evolutionary history was inferred by using the Maximum Likelihood method based on the General Time Reversible model [89]. The tree with the highest log likelihood (-25430.37) is shown. Initial tree(s) for the heuristic search were obtained automatically by applying Neighbor-Join and BioNJ algorithms to a matrix of pairwise distances estimated using the Maximum Composite Likelihood (MCL) approach, and then selecting the topology with superior log likelihood value. The tree is drawn to scale, with branch lengths measured in the number of substitutions per site. The analysis involved 77 nucleotide sequences. Codon positions included were 1st+2nd+3rd. There were a total of 2427 positions in the final dataset. Evolutionary analyses were conducted in MEGA7 [87]. (TIF) [file pone.0252365.s009.tif]
