# Supplementary material for: Fusarium graminearum DICER-like-dependent sRNAs are required for the suppression of host immune genes and full virulence
Source: PLoS One. 2021 Aug 5;16(8):e0252365. doi: 10.1371/journal.pone.0252365 (PMC8341482; doi:10.1371/journal.pone.0252365)
Supplement: S10 Fig — (PDF) [file pone.0252365.s010.pdf]

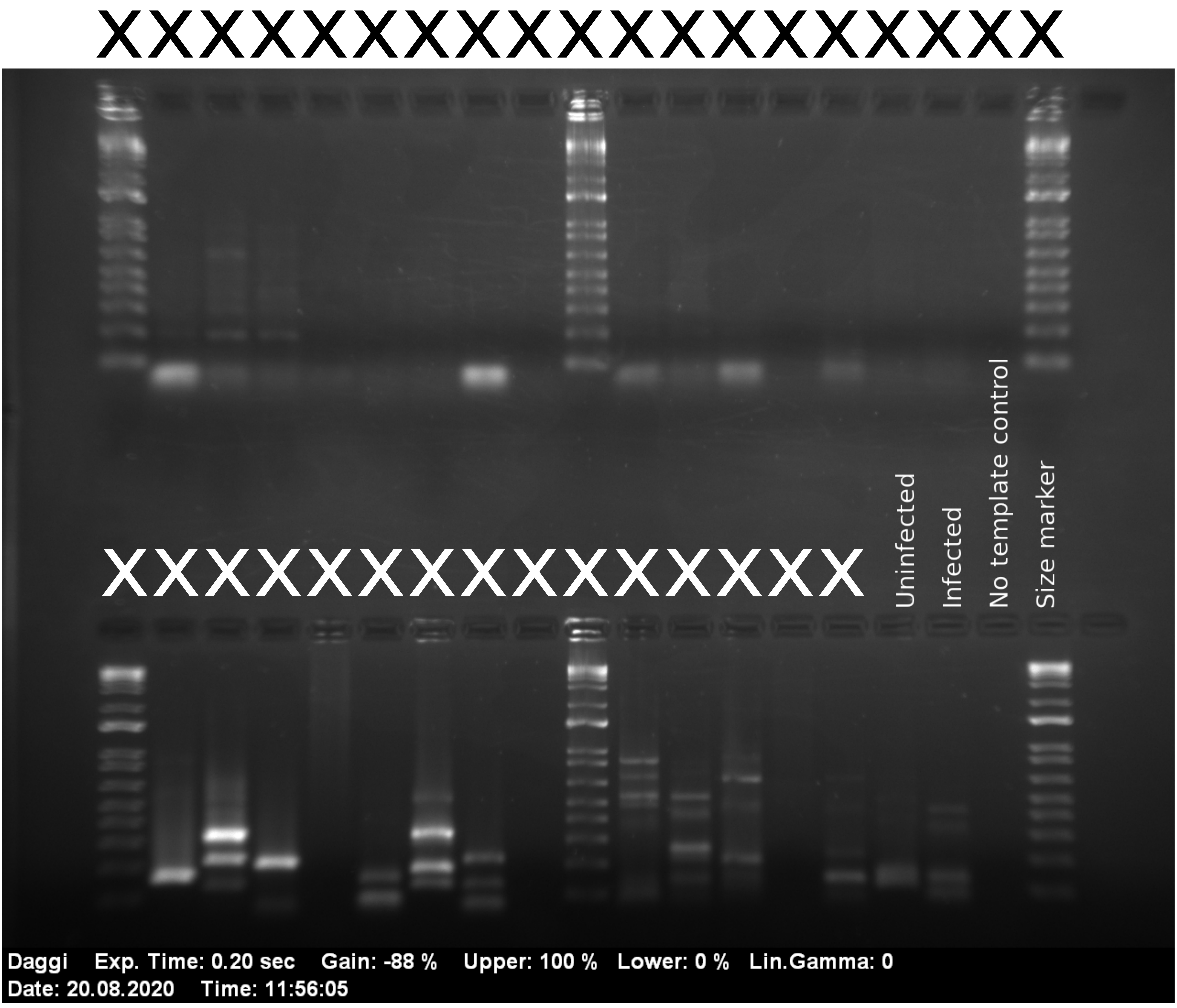

Fig. 6D

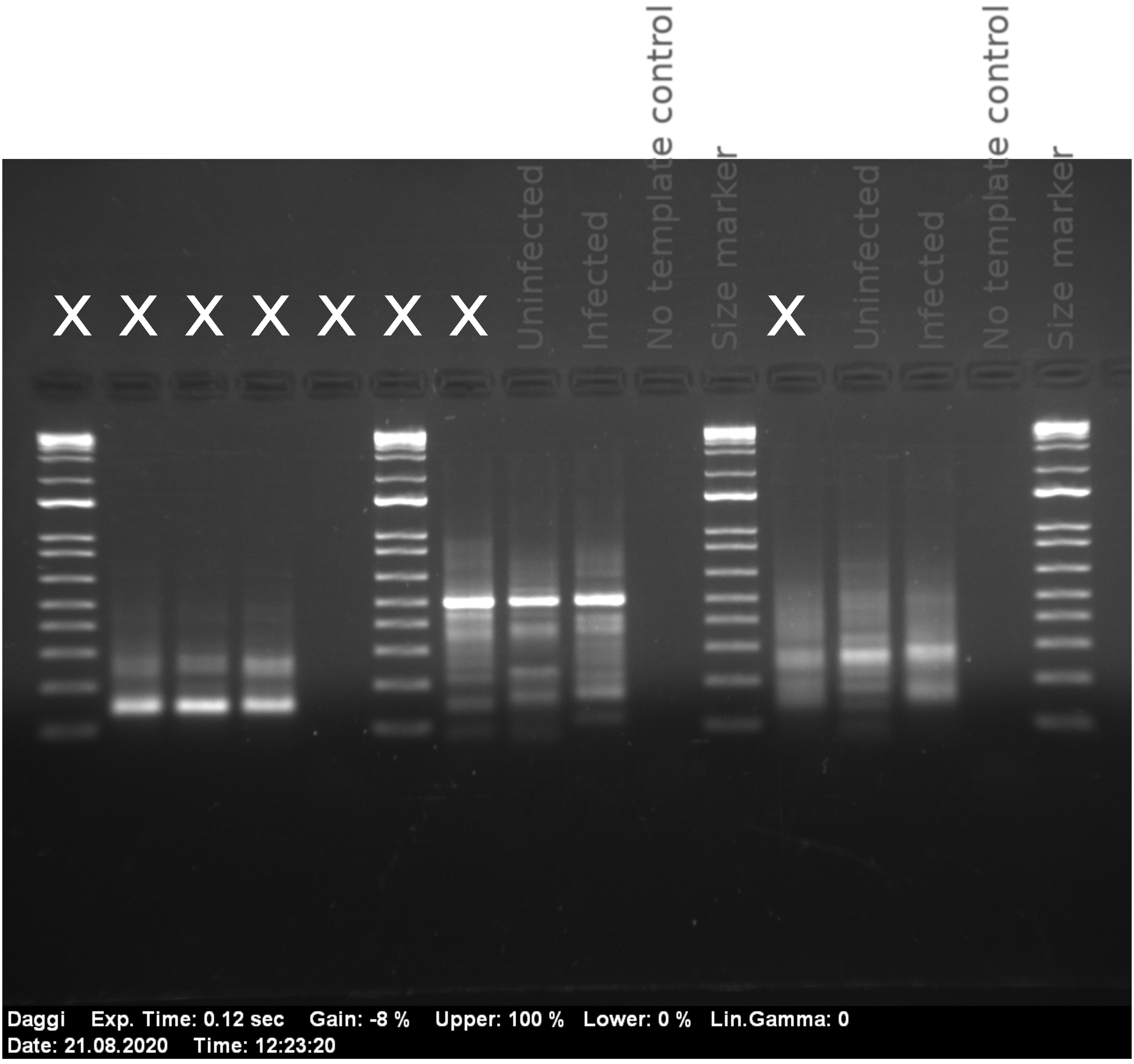

Fig. 6E Fig. 6F

Products of the RLM-RACE-PCR were run on a 1.5% agarose gel stained with EtBr.
