# Supplementary material for: Fusarium graminearum DICER-like-dependent sRNAs are required for the suppression of host immune genes and full virulence
Source: PLoS One. 2021 Aug 5;16(8):e0252365. doi: 10.1371/journal.pone.0252365 (PMC8341482; doi:10.1371/journal.pone.0252365)
Supplement: S1 Table — (DOCX) [file pone.0252365.s011.docx]

## S1 Table. Primer sequences.

| Gene | Accession | Primer Name | Sequence |
| --- | --- | --- | --- |
| *HvARF3* | HORVU1Hr1G076690 | HORVU1Hr1G076690_F | GGTTCAGCTCAGAAACGAAGC |
|  |  | HORVU1Hr1G076690_R | ATTCTGACGCTCCACTCCTTG |
| *HvPPR* | HORVU2Hr1G078260 | HORVU2Hr1G078260_F | GGGTGCTTCATCGAGTTGGAA |
|  |  | HORVU2Hr1G078260_R | CTGCAAAACCACAGAGCTTGT |
| *HvSERK2* | HORVU2Hr1G080020 | HORVU2Hr1G080020.6_F | GATGACAGACAGAGTCCTGCT |
|  |  | HORVU2Hr1G080020.6_R | AGCACTACTACCAGCACCGA |
| *HvARF10* | HORVU2Hr1G089670 | HORVU2Hr1G089670_F | CACATCGGCGATGAACCTTTC |
|  |  | HORVU2Hr1G089670_R | TCGGCTCAAGATCGATGGATG |
| *HvPGLP2* | HORVU5Hr1G052320 | HORVU5Hr1G052320_F | CTCCTTGTTCTGTCAGGTGTGA |
|  |  | HORVU5Hr1G052320_R | ATTGCTGGTGCTGTATTCGGA |
| *HvATG2* | HORVU6Hr1G034660 | HORVU6Hr1G034660_F | TTCTTATCTCGGGGCTTGGTG |
|  |  | HORVU6Hr1G034660_R | TCGTAGCAGCCAAGAACCATT |
| *HvGDH* | HORVU6Hr1G076880 | HORVU6Hr1G076880_F | GGCAACGTGGAGAGTGTGAA |
|  |  | HORVU6Hr1G076880_R | GTACGGGCTCGAGTTGATCAG |
| *HvARF19* | HORVU7Hr1G096460 | HORVU7Hr1G096460_F | GGGCCGGTCTATCGACATTAG |
|  |  | HORVU7Hr1G096460_R | TTGACAAACTCCTCCCAAGGG |
| *HvSUB1* | HORVU2Hr1G028070 | MLOC_12796.1_F | CAGAGTTCAGGAGGGGCAAG |
|  |  | MLOC_12796.1_R | GACAAACGTCCGGTTGAGGA |
| *HvSUVR5* | HORVU6Hr1G069350 | MLOC_14605.1_F | TGCATTTTGTTGACCGCAGG |
|  |  | MLOC_14605.1_R | AGGCTTGTCTGGGAACGATG |
| *Hvemb2726* | HORVU5Hr1G024470 | MLOC_58105.1_F | AGACTGATGTTGCGGTGGAG |
|  |  | MLOC_58105.1_R | GGTTGCGACCTAACTTGGGA |
| *HvPIX7* | HORVU3Hr1G051080 | MLOC_5991.1_F | GATGGGCTTCAGGGGCATAA |
|  |  | MLOC_5991.1_R | ATGGGAGCGGAAATGACCTC |
| *HvRDR1* | HORVU6Hr1G074180 | MLOC_75294.1_F | TATCTGAAGGTTCGGCCTGC |
|  |  | MLOC_75294.1_R | GTTCCGCTCCACAGAACAGA |
| *HvRST1* | HORVU3Hr1G016630 | MLOC_75306.1_F | TTGCGGGACTTGTTCTTGGT |
|  |  | MLOC_75306.1_R | TGACAGATGGCAGAGCAAGG |
| *HvEOL1* | HORVU2Hr1G119180 | MLOC_8741_F | CACTTCAAGCCCGCTGACTA |
|  |  | MLOC_8741_R | CTCATGTATCGTGCTCGCCT |
| *BdSERK2* | BRADI_5g12227v3 | PNT61220_F | AGTTGCGTTTCCTCCGTCTT |
|  |  | PNT61220_R | ACCAGTTGATGGAACCTCTCC |
| *HvUBI* | HORVU1Hr1G023660 | Ubideg60_F | ACCCTCGCCGACTACAACAT |
|  |  | Ubideg60_R | CAGTAGTGGCGGTCGAAGTG |
| *FgEF1a* | FGSG_08811 | EF1a_F | CAAGGCCGTCGAGAAGTCCAC |
|  |  | EF1a_R | TGCCAACATGATCATTTCGTCGTA |
| Name | **Sequence (RNA)** | **Primer** | **Sequence (Primer)** |
| *Hvu*-miRNA-159a | UUUGGAUUGAAGGGAGCUCUG | hvu-mir159a_F | TGGCTCGCTtttggattgaaggga |
|  |  | hvu-mir159a_RT | GTCGTATCCAGTGCAGGGTCCGA  GGTATTCGCACTGGATACGACcagagc |
| *Hvu*-miRNA-168 | UCGCUUGGUGCAGAUCGGGAC | hvu-mir168-5p_F | GTTCGCTtcgcttggtgcagat |
|  |  | hvu-mir168-5p_RT | GTCGTATCCAGTGCAGGGTCCGA  GGTATTCGCACTGGATACGACgtcccg |
| *Fg*-sRNA-321 | GCUUGGGUCCCGAGGGGCUACC | Fg-sRNA_321-2106_F | TCGCTccatcggggagccctg |
|  |  | Fg-sRNA_321-2106_RT | GTCGTATCCAGTGCAGGGTCCGA  GGTATTCGCACTGGATACGACcgaacc |
| *Fg*-sRNA-1921 | CUUGGGUCCCGAGGGGCUACC | Fg-sRNA_1921-416_F | TCGCTccatcggggagccct |
|  |  | Fg-sRNA_1921-416_RT | GTCGTATCCAGTGCAGGGTCCGA  GGTATTCGCACTGGATACGACgaaccc |
| *Fg*-sRNA-6717 | UAGCUUGGGUCCCGAGGGGCUAC | Fg-sRNA_6717-86_F | TCGCTcatcggggagccctggg |
|  |  | Fg-sRNA_6717-86_RT | GTCGTATCCAGTGCAGGGTCCGA  GGTATTCGCACTGGATACGACatcgaa |
| Universal SL Reverse |  | UniSL_R | CCAGTGCAGGGTCCGAGGTA |
| Target | **Accession** | **Name** | **Sequence** |
| RLM-adapter | | RLM_Adapter | GCUGAUGGCGAUGAAUGAACACUG  CGUUUGCUGGCUUUGAUGAAA |
| RLM outer adapter Primer | | RLM_Uni_O1 | GCTGATGGCGATGAATGAACACTG |
| RLM inner adapter primer | | RLM_Uni_I1 | GAACACTGCGTTTGCTGGCTTTGATG |
| *HvEOL1* | HORVU2Hr1G119180 | HvEOL1_outer | GAATTTACTGATGGCCCGCAT |
|  | HORVU2Hr1G119180 | HvEOL1_inner | ACCCACCATTAAGCATCGCA |
| *HvSERK2* | HORVU2Hr1G080020 | HvSERK2_1_outer | GAGCCTCAGGAGACGGTTTT |
|  | HORVU2Hr1G080020 | HvSERK2_1_inner | AGTGGAGTCGACGATCCAGT |
| *HvBAK1* | HORVU7Hr1G068990 | HvSERK2_2_outer | GGGTTTGCACATGCTCGTAC |
|  | HORVU7Hr1G068990 | HvSERK2_2_inner | TGAGGACCCAGCTCTACCTC |

Sequences and target accessions for all primers used in the study
